# Cardiac Troponin I as a Prognostic Indicator of Mortality in Patients With Sepsis and Organ Dysfunction

**DOI:** 10.7759/cureus.101641

**Published:** 2026-01-15

**Authors:** Mahrukh LNU, Jamil Muqtadir, Imtiaz Begum, Irshad Batool, Sameeullah Bhatti, Sidra Khan, Abdur Rahman Ansari, Muhammad Tahseen, Abdul Rasheed, Ahmed Wahab

**Affiliations:** 1 Internal Medicine, Dr. Ziauddin Hospital, Karachi, PAK; 2 Infectious Diseases, Dr. Ziauddin Hospital, Karachi, PAK; 3 Gastroenterology and Hepatology, Isra University Hospital, Hyderabad, PAK; 4 Gastroenterology, Aga Khan University Hospital, Karachi, PAK; 5 Intensive Care Medicine, Mount Sinai Hospital, New York City, USA; 6 General Medicine, Dr. Ziauddin Hospital, Karachi, PAK; 7 Medicine, Dr. Ziauddin Hospital, Karachi, PAK; 8 Cardiology, National Institute of Cardiovascular Diseases, Karachi, PAK

**Keywords:** coagulopathy, icu, inflammation, mortality, organ dysfunction, prognostic biomarker, sepsis, troponin

## Abstract

Introduction and aim

Sepsis is a complex, life-threatening condition characterized by systemic inflammation and multiorgan dysfunction. Elevated troponin levels (cTnI) have been associated with poor outcomes in sepsis; however, their role as a prognostic biomarker requires further investigation. This study aimed to (1) determine the association between elevated cardiac troponin I (>0.04 ng/mL) and 28-day mortality in patients with sepsis and organ dysfunction, and (2) evaluate the prognostic accuracy of troponin I for mortality prediction in this population.

Methods

We conducted a prospective cohort study involving 200 sepsis patients, measuring troponin levels at admission time and clinical and biochemical parameters, such as Sequential Organ Failure Assessment (SOFA) scores, D-dimer, and lactate. Data on organ dysfunction, coagulation abnormalities, and patient outcomes (mortality, length of ICU stay) were collected and analyzed.

Results

Troponin I levels were elevated in 69% of patients and were significantly associated with increased mortality (115 {83.4%} vs. 10 {16.2%}; p<0.001), longer ICU duration, and higher SOFA scores. Multivariate analysis revealed that elevated creatinine, low hemoglobin, increased CRP and alanine transaminase (ALT), neutrophilia, and reduced oxygen saturation were independent predictors of 28-day mortality. Troponin I showed high prognostic accuracy for mortality, and a cut-off value of >0.04 ng/mL demonstrated remarkable accuracy, achieving an AUC of 0.986 in receiver operating characteristic (ROC) analysis.

Conclusion

Troponin I serves as a readily accessible and prognostically significant biomarker that improves early risk assessment in patients experiencing sepsis with organ dysfunction.

## Introduction

Sepsis is characterized as a life-threatening organ failure resulting from a dysregulated host response to infection, as specified in the Sepsis-3 consensus guidelines [1]. Troponin I (cTnI), a cardiac-specific protein that regulates myocardial contraction, is among the emerging biomarkers for this purpose [2]. Cardiac muscle troponin I, a myofibrillar regulatory protein, is a sensitive and specific marker of myocardial injury [3]. Elevation of troponin I in sepsis is not solely attributable to ischemia; it may also arise from myocardial strain, the release of inflammatory cytokines, microvascular dysfunction, or direct injury to cardiomyocytes, even when coronary artery occlusion is not present [4].

Multiple studies have reported an association between elevated troponin I and increased mortality. In the ICU setting, troponin I measurement can add considerable value to clinicians in assessing patients at higher risk of adverse outcomes so that more intensive monitoring and intervention can be employed [5-8]. Elevated troponin I levels have been associated with more severe organ function, longer stays in the ICU, and a greater need for the administration of vasopressors [9]. Consistent with an association with increased mortality, elevated troponin I has been used as a valuable tool for risk stratification in critically ill patients. The value of troponin I in guiding therapy is still not well established, whereas the prognostic value of troponin I in sepsis is well established by current evidence. The primary objectives were to assess the association between troponin I elevation and 28-day mortality and to evaluate the prognostic performance of troponin I for mortality prediction in sepsis with organ dysfunction. Secondary objectives were to explore associations of troponin I with markers of organ dysfunction and coagulation abnormalities.

## Materials and methods

Study design and sample size

This prospective study was performed in the Department of General Medicine at Dr. Ziauddin Hospital, Karachi, Pakistan, between April 2024 and May 2025. This study was approved by the Research Department of the College of Physicians and Surgeons, Pakistan (CPSP), and the Ethics Review Committee of Dr. Ziauddin University.

Inclusion and exclusion criteria

The patients were included in the study if they met the following criteria: patients aged 18-65 years of both genders, patients admitted with sepsis and organ dysfunction as defined by the Sepsis-3 criteria (Sequential Organ Failure Assessment {SOFA} score ≥2) [1]. Individuals with a documented history of acute coronary syndrome in the last 30 days were excluded to minimize confounding factors related to primary cardiac injury. Individuals with end-stage renal disease undergoing dialysis were also excluded. All comorbidities, including diabetes, hypertension, chronic liver disease, and controlled cardiac arrhythmias, were included to accurately represent real-world internal medicine populations.

Sample and data collection

Patients were screened for sepsis and organ dysfunction. Patients were considered to have sepsis with organ dysfunction meeting both initial screening with Systemic Inflammatory Response Syndrome (SIRS) criteria as follows: a body temperature of >38°C or <36°C, a heart rate of >90 beats/min, a respiratory rate of >20 breaths/min, or a partial pressure of CO_2_ of <32 mmHg [1], and additionally, were characterized by at least one organ dysfunction, including hypotension systolic blood pressure (SBP) <90 mmHg or mean arterial pressure (MAP) of <65 mmHg; creatinine >2.0 mg/dL or urine output <0.5 mL/kg/h x 2 h; bilirubin >2.0 mg/dL; platelet count <100,000/mm^3^; international normalized ratio (INR) >1.5 or partial thromboplastin time (PTT) >60 s; and lactate >2 mmol/L [10]. Upon confirmation of sepsis with organ dysfunction, the study objectives were explained to patients or their immediate relatives, and written informed consent was obtained. Then, 5 mL of blood was drawn using an aseptic technique for measuring troponin I levels. Troponin assays used in this cohort corresponded to the high-sensitivity troponin assays. Troponin I levels were assessed within the initial 6 h of admission to maintain uniformity in timing among patients. Elevated troponin I was characterized by a serum level exceeding 0.04 ng/mL, according to the reference range established by the institutional laboratory for myocardial injury. Echocardiography was performed according to clinical criteria and within 8 h of troponin measurement. All patients received standard sepsis treatment, including broad-spectrum antibiotics, fluid resuscitation, vasopressors, and other supportive care as required, based on their clinical condition. All patients were followed for 28 days to assess the outcome variable (mortality). At enrollment, baseline demographic details, including age, gender, weight, height, body mass index (BMI), and smoking status, were recorded. Clinical details, including a history of chronic conditions (diabetes or hypertension), previous ICU admissions, and organ function assessments, were documented.

Statistical analysis

No a priori sample size calculation was performed because this was a consecutive observational cohort during the study period; analyses emphasize effect estimates and confidence intervals. The data's normality was determined using the Shapiro-Wilk test at p>0.05. Qualitative variables, including gender, residential status, diabetes mellitus, hypertension, smoking status, the need for ventilation, type of ventilation, need for inotropic support, and mortality, were presented as frequency and percentage. Quantitative variables, including age, creatinine, bilirubin, platelets, duration of ICU stay, and hospital stay, are reported as mean and SD or median (IQR) as appropriate. Effect modifiers/confounders, including age, gender, residential status, diabetes mellitus, hypertension, smoking status, need for ventilation, type of ventilation, need for inotropic support, duration of ICU stay, and length of hospital stay, were controlled through stratification. For post-stratification, an appropriate chi-square/Fisher's Exact test was used. Relative risk (RR) with 95% CI was calculated. Subgroup analysis was performed using the Mann-Whitney U test or one-way ANOVA as appropriate. Pearson’s correlational analysis determined the correlation between troponin I levels and biochemical variables. Multivariate logistic regression analysis determined the factors associated with 28-day mortality. Receiver operating characteristic (ROC) analysis was performed to determine the prognostic value of troponin as an indicator of mortality. The multivariable model was constructed to identify independent clinical predictors of mortality; troponin I was analyzed as a stratification variable. ROC analysis evaluated the discriminative ability for 28-day mortality using admission troponin I, defined by the institutional threshold (>0.04 ng/mL), and reported the AUC with 95% confidence intervals. Survival analysis was performed to see the prognostic ability of troponin for 28-day mortality. For all statistical analyses, p<0.05 was considered statistically significant. All data analyses were performed on GraphPad Prism version 9.0 (Boston, MA: GraphPad Software). Analyses excluded cases with missing data for key variables.

## Results

Basic data characteristics and their association with outcome (death)

**Figure 1:**
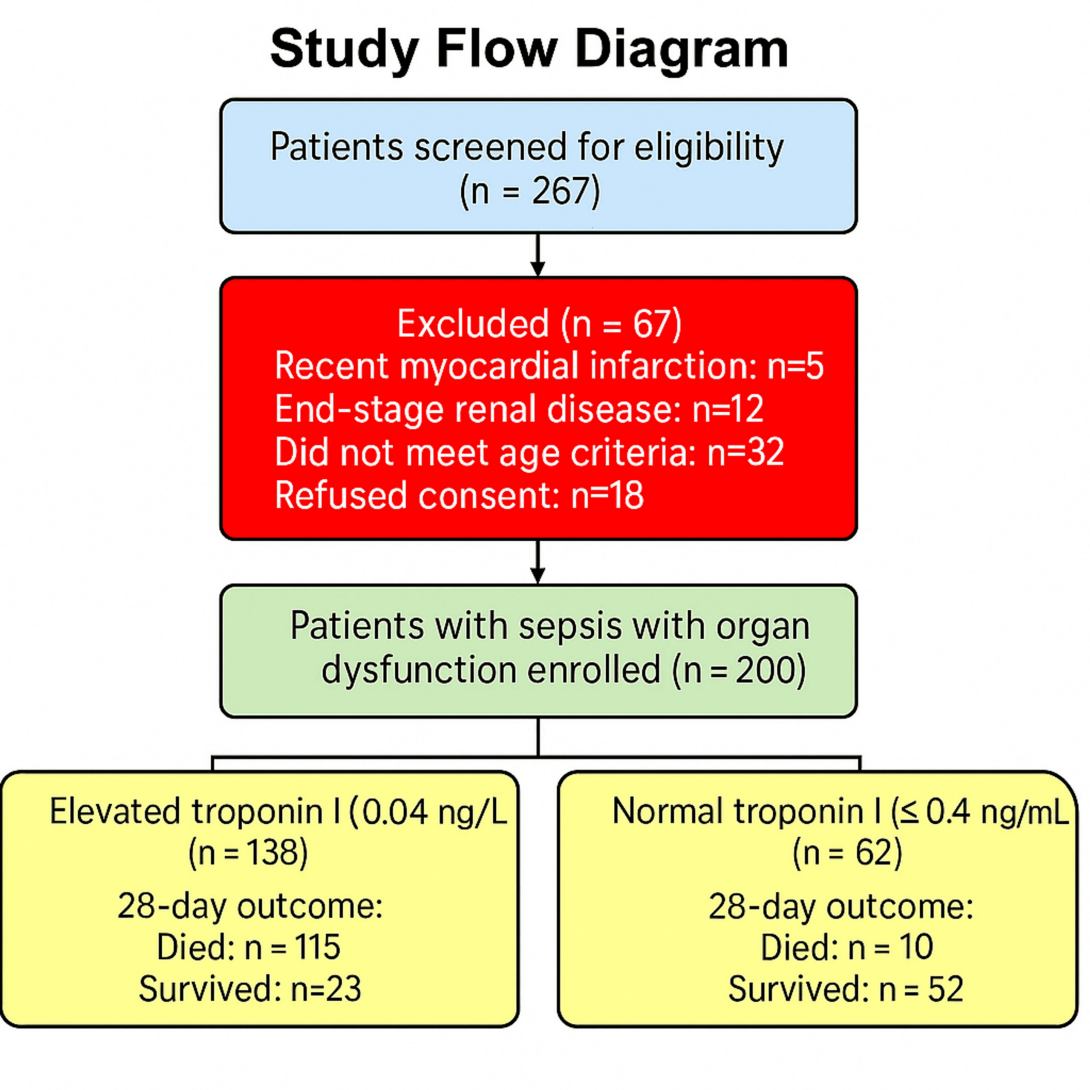
Flow diagram of the study cohort.

Figure 1 presents the participant flow diagram illustrating screening, exclusions, and the final analytic cohort. Out of the 200 patients enrolled, 54.5% (109) were female, with a mean age of 47.8±22.2 years (Table 1). Troponin I levels were elevated in 69% (138) of patients. The mortality rate in this group was markedly elevated at 83.4% (115), compared with 16.2% (10) in individuals with normal troponin levels (RR=5.74, p<0.001). Individuals diagnosed with cardiovascular disease (CVD) exhibited a significantly elevated prevalence of abnormal troponin levels (12.3% {17/138} vs. 1.6% {1/62}, RR=7.55, p=0.0001). Furthermore, abnormal echocardiograms were observed in 73.2% (101) of patients with elevated troponin compared with 24.1% (15) in the normal group (p<0.001), and arrhythmia occurred more frequently in the elevated group (90 {65.2%} vs. 15 {24.1%}, p=0.0004). No notable variation in troponin levels was detected among the different sources of infection.

**Table 1:** Basic demographic data and relative risk associated with abnormal troponin I level in patients with sepsis (n=200). CVD: cardiovascular diseases; COPD: chronic obstructive pulmonary disease

Variables	Categories	Abnormal (n=138) (%)	Normal (n=62) (%)	Relative risk	p-Value
Gender	Female	77 (55.7)	32 (51.6)	1.05	0.582
	Male	61 (44.3)	30 (48.4)		
Outcome	Died	115 (83.4)	10 (16.2)	5.74	<0.001
	Survived	23 (16.6)	52 (83.8)		
Pre-existing conditions	CVD	17 (12.3)	1 (1.6)	7.55	0.134
	Diabetes	32 (23.1)	16 (25.8)	0.89	0.683
	Hypertension	22 (15.9)	13 (20.9)	0.76	0.426
	COPD	10 (7.2)	7 (11.2)	0.64	0.346
	None	57 (41.3)	25 (40.3)	1.01	1.00
Echocardiography	Normal	37 (26.8)	47 (75.8)	2.012	<0.001
Arrhythmia	Yes	90 (65.2)	15 (24.1)	1.168	0.135
Infection source	Abdomen	30 (21.7)	22 (35.4)	0.61	0.055
	Bloodstream	20 (14.5)	8 (12.9)	1.12	0.829
	Lung	57 (41.3)	19 (30.6)	1.35	0.160
	Other	7 (5.0)	4 (6.4)	0.79	0.741
	Urinary	24 (17.3)	9 (14.5)	1.20	0.685
Vasopressor use	Yes	72 (52.1)	27 (43.5)	1.158	0.259
Mechanical ventilation	Yes	83 (60.1)	36 (58.0)	1.201	0.781
Steroid use	Yes	57 (41.3)	36 (58.0)	1.58	0.027

Association of clinical parameters with abnormal troponin I levels

**Figure 2:**
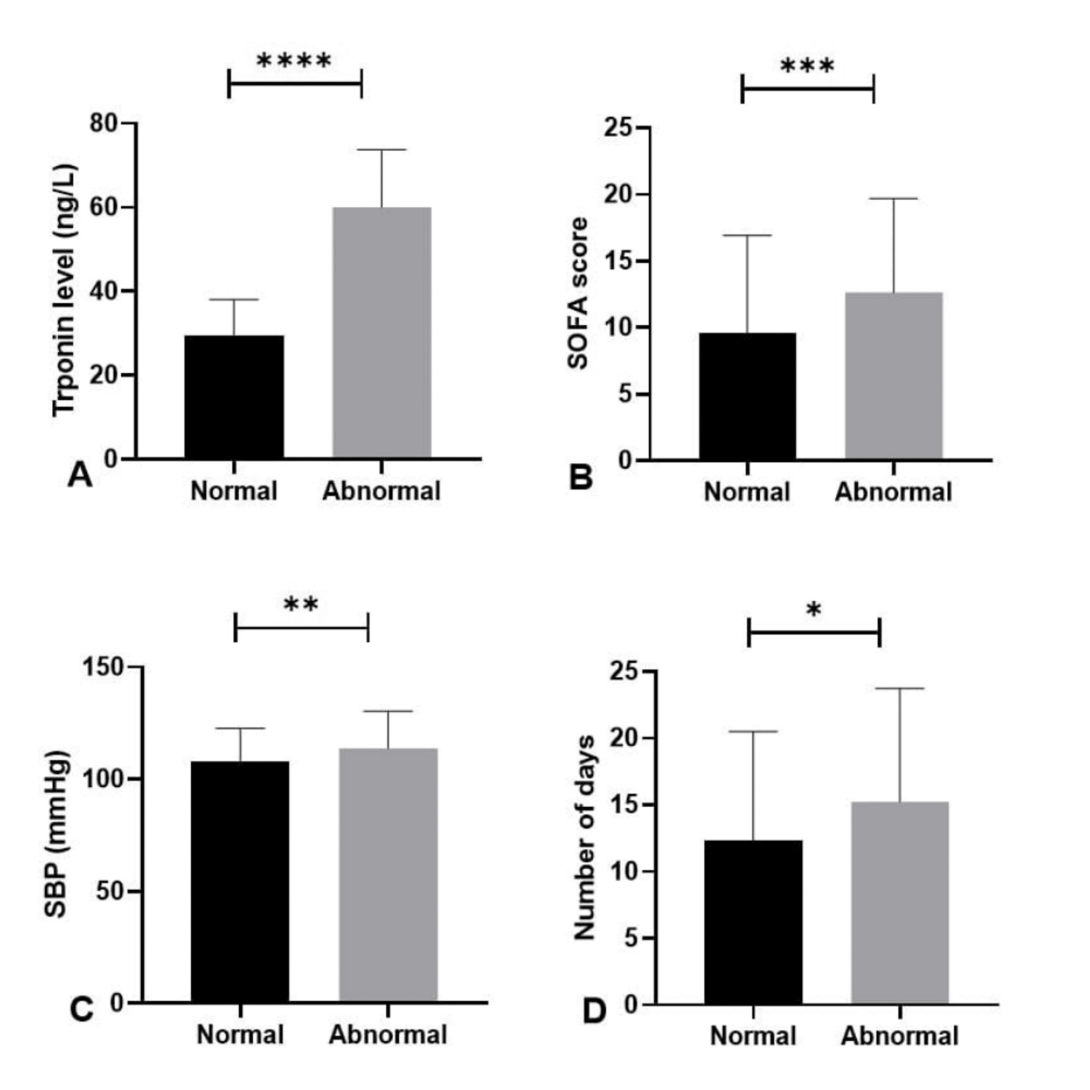
Comparison of clinical parameters between sepsis patients with normal and elevated troponin I levels. *P<0.05 was statistically significant. **P<0.01 was statistically significant. ***P<0.001 was statistically significant. ****P<0.0001 was statistically significant. The bar graph represents the mean value, and the error bar represents the standard deviation. Normal troponin levels are black bars; grey bars represent abnormal troponin I levels. (A) Comparison of troponin levels, (B) comparison of SOFA score, (C) comparison of systolic BP, and (D) comparison of ICU stay. The comparison was made through the Mann-Whitney U test. P<0.05 was considered statistically significant. SOFA: Sequential Organ Failure Assessment; SBP: systolic blood pressure

The clinical and biochemical parameters between sepsis patients with normal and elevated troponin levels were compared at admission (Table 2). Elevated troponin levels were observed in most patients (Figure 2A). Patients exhibiting elevated troponin levels demonstrated significantly higher SOFA scores (12.64±7.02 compared to 9.61±7.32, p=0.005) (Figure 2B). The elevated group exhibited a higher systolic blood pressure (113.8±16.6 vs. 108±14.7 mmHg, p=0.003), likely attributable to vasopressors (Figure 2C), and extended ICU stays (15.2±8.5 days vs. 12.3±8.1 days, p=0.026) (Figure 2D). No notable differences were detected in BMI, heart rate, respiratory rate, temperature, or duration of hospital stay among the groups.

**Table 2:** Comparison of clinical and biochemical parameters between sepsis patients with normal and abnormal troponin I levels (n=200). SOFA: Sequential Organ Failure Assessment; BP: blood pressure; BMI: body mass index; ICU: intensive care unit; HB: hemoglobin; WBC: white blood cells; RBC: red blood cells; BUN: blood urea nitrogen; ALT: alanine transaminase; AST: aspartate transaminase; CRP: C-reactive protein; PaO_2_: partial pressure of oxygen in arterial blood; LDH: lactate dehydrogenase; APTT: activated partial thromboplastin time; PT: prothrombin time; n: number; SD: standard deviation

Parameters	Abnormal (n=138)	Normal (n=62)	p-Value
	Mean±SD	Mean±SD	
SOFA score	12.64±7.02	9.613±7.32	0.005
BP	113.81±6.64	108±14.76	0.003
BMI	24.99±5.15	25.58±4.51	0.411
Heart rate	101.2±27.31	105.5±28.1	0.353
Respiratory rate	20.46±5.24	20.81±5.12	0.613
Temperature	37.47±1.25	37.46±1.22	0.754
Oxygen saturation	94.84±2.63	95.49±2.57	0.093
Fluid resuscitation	2.412±0.85	2.51±0.83	0.612
ICU stay	15.23±8.51	12.31±8.19	0.026
Hospital stay	33.6±15.98	32.03±16.33	0.531
HB	10.84±2.26	12.43±2.09	<0.001
Hematocrit	39.52±4.35	41.93±4.84	0.001
WBC	8.81±3.04	7.536±2.64	0.021
Platelets	258.9±49.71	241.5±50.92	0.010
RBC count	4.472±0.51	4.54±0.51	0.431
Neutrophils	74.74±9.59	61.64±9.83	<0.001
Lymphocytes	26.58±5.27	29.07±5.84	0.004
Prolactin	1.02±0.45	0.86±0.54	0.029
BUN	20.29±4.98	18.65±4.35	0.013
Creatinine	1.01±0.29	0.87±0.34	0.002
ALT	42.82±15.38	35.57±13.47	0.002
AST	37.16±12.12	31.66±11.53	0.003
D-dimers	1.09±0.43	0.88±0.41	0.001
CRP	60.37±19.58	48.08±16.74	<0.001
PaO_2_	80.53±20.02	80.8±23.84	0.858
LDH	2.33±0.95	1.74±0.99	<0.001
Ferritin	361.6±113.4	271.1±103.7	<0.001
APTT	31.6±5.43	29.1±4.63	0.002
PT	11.96±2.07	10.53±2.96	<0.001

**Figure 3:**
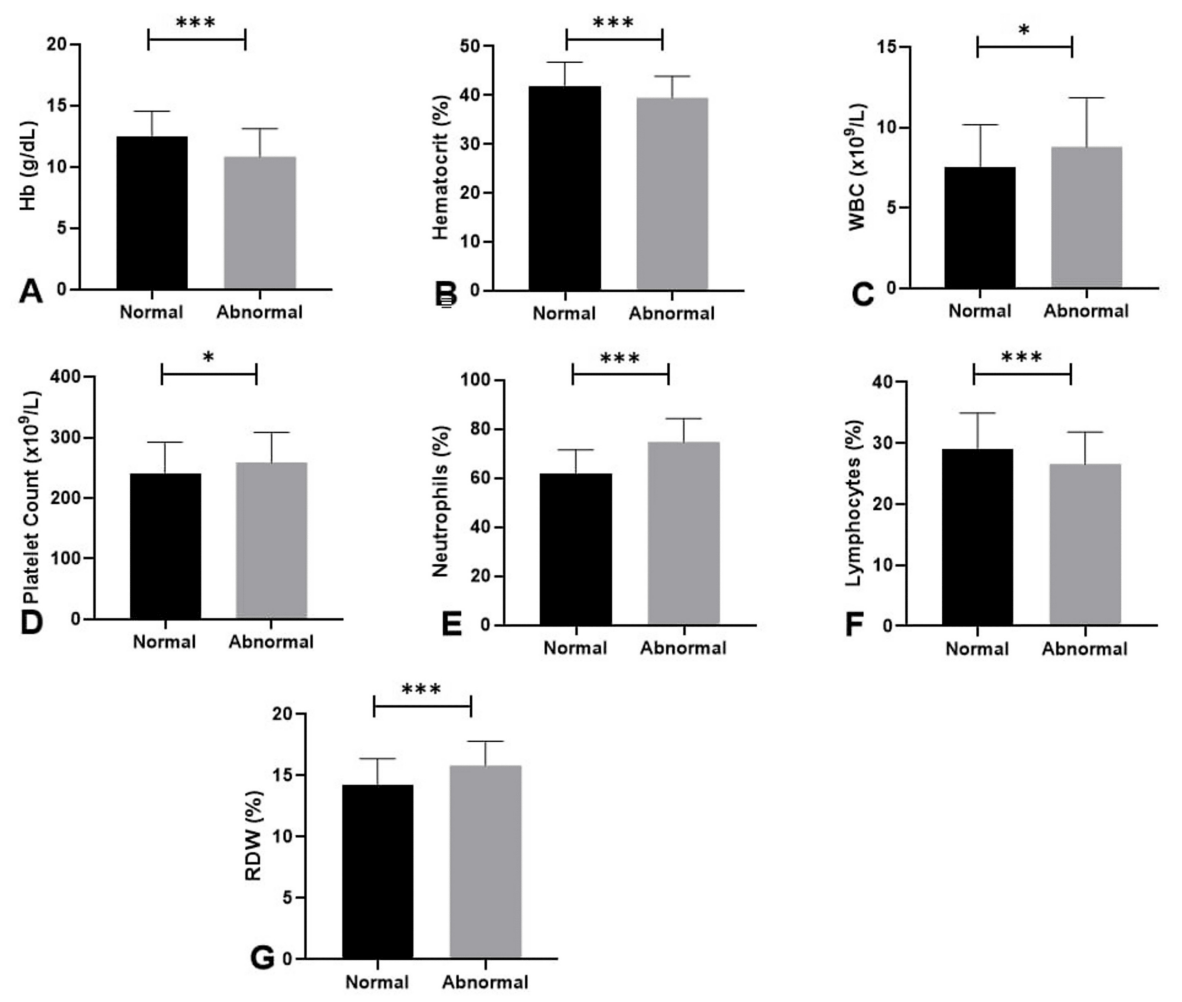
Comparison of blood parameters between sepsis patients with normal and abnormal troponin I levels. *P<0.05 was statistically significant. **P<0.01 was statistically significant. ***P<0.001 was statistically significant. ****P<0.0001 was statistically significant. The bar graph represents the mean value, and the error bar represents the standard deviation. Normal troponin levels are black bars; grey bars represent abnormal troponin I levels. (A) Comparison of HB, (B) comparison of hematocrit (%), (C) comparison of WBC count, (D) comparison of platelet count, (E) comparison of neutrophils (%), (F) comparison of lymphocytes (%), and (G) comparison of RDW (%). The comparison was made through the Mann-Whitney U test. P<0.05 was considered statistically significant. Hb: hemoglobin; WBC: white blood cells; RDW: red cell distribution width

Significant differences were noted in hematological parameters. Patients displaying abnormal troponin levels had significantly lower hemoglobin levels (10.84±2.26 vs. 12.43±2.09, p<0.001) and decreased hematocrit values (39.5% vs. 41.9%, p=0.001) (Figures 3A, 3B). The white blood cell (WBC) count was notably greater in the elevated group (8.81±3.04 vs. 7.53±2.64, p=0.021) (Figure 3C). Patients with elevated troponin exhibited higher platelet counts (258.9±49.7 vs. 241.5±50.9, p=0.01) (Figure 3D). The neutrophil percentage was significantly increased (74.7% vs. 61.6%, p<0.001), whereas the lymphocyte percentage was decreased (26.6% vs. 29.1%, p=0.004) in this cohort (Figures 3E, 3F). Red cell distribution width (RDW) percentage was significantly elevated in the abnormal troponin group (15.83±1.96 vs. 14.21±2.16, p<0.001), indicating potential erythropoietic stress or anisocytosis in critically ill patients (Figure 3G).

**Figure 4:**
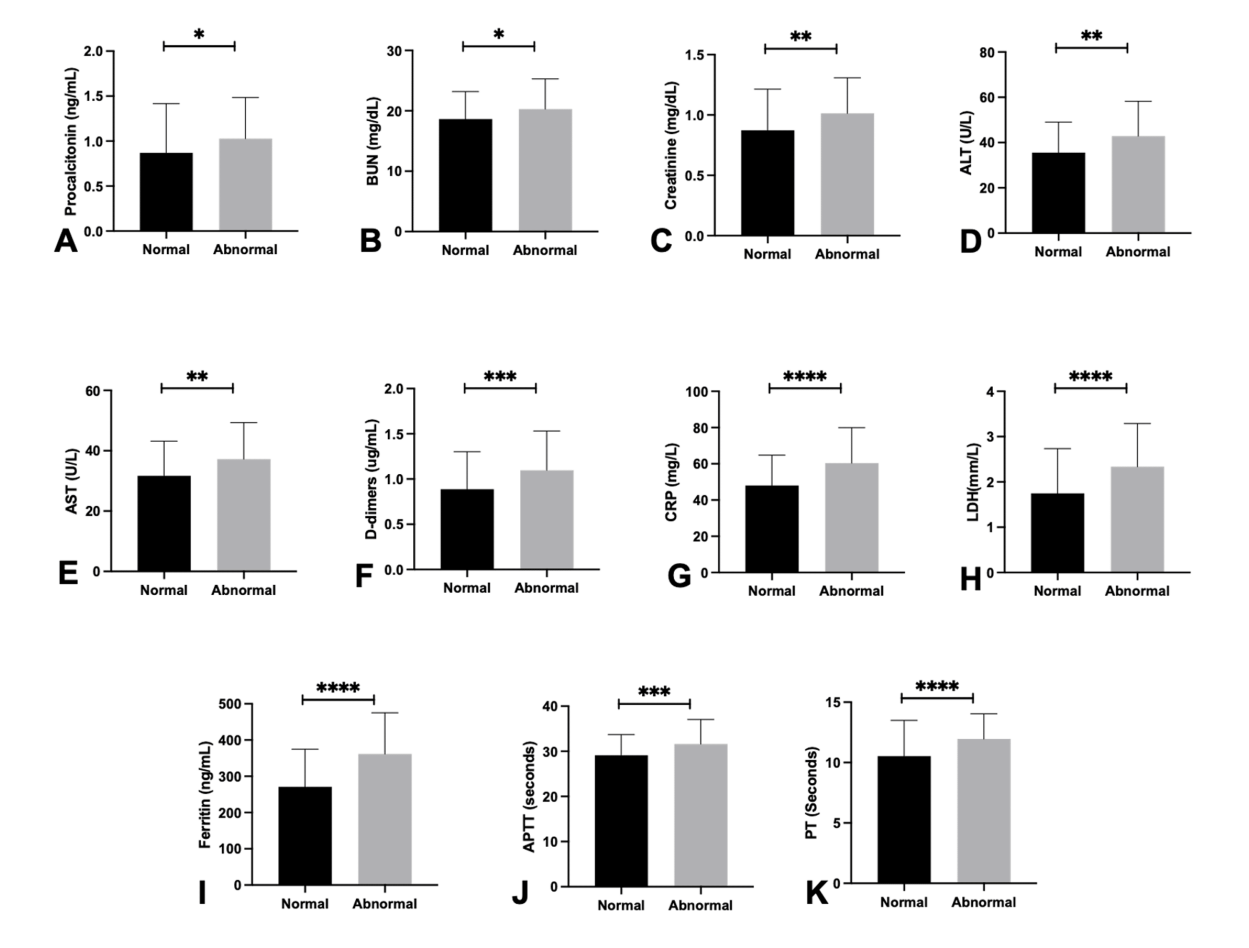
Comparison of biochemical parameters between sepsis patients with normal and abnormal troponin I levels. *P<0.05 was statistically significant. **P<0.01 was statistically significant. ***P<0.001 was statistically significant. ****P<0.0001 was statistically significant. The bar graph represents the mean value, and the error bar represents the standard deviation. Normal troponin levels are black bars; grey bars represent abnormal troponin I levels. (A) Comparison of procalcitonin, (B) comparison of BUN, (C) comparison of creatinine, (D) comparison of ALT, (E) comparison of AST, (F) comparison of d-dimers, (G) comparison of CRP, (H) comparison of LDH, (I) comparison of ferritin, (J) comparison of APTT, and (K) comparison of PT. The comparison was made through the Mann-Whitney U test. P<0.05 was considered statistically significant. BUN: blood urea nitrogen; ALT: alanine transaminase; AST: aspartate transaminase; CRP: C-reactive protein; LDH: lactate dehydrogenase; APTT: activated partial thromboplastin time; PT: prothrombin time

The relation of troponin with biochemical parameters is shown in Figures 4A-4K. Numerous biochemical markers showed significant elevation in patients presenting with elevated troponin levels. The results indicated significant differences in procalcitonin levels (1.02±0.45 vs. 0.86±0.54, p=0.029), blood urea nitrogen (BUN) (20.3±5.0 vs. 18.7±4.4, p=0.013), creatinine (1.01±0.29 vs. 0.87±0.34, p=0.002), alanine transaminase (ALT) (42.8±15.4 vs. 35.6±13.5, p=0.002), and aspartate transaminase (AST) (37.2±12.1 vs. 31.7±11.5, p=0.003). Coagulation markers, including D-dimer (1.09±0.43 vs. 0.88±0.41, p=0.001), prothrombin time (PT) (11.96 vs. 10.53 s, p<0.001), and activated partial thromboplastin time (APTT) (31.6 vs. 29.1 s, p=0.002), were significantly elevated. CRP (60.4±19.6 vs. 48.1±16.7, p<0.001), lactate dehydrogenase (LDH) (2.33±0.95 vs. 1.74±0.99, p<0.001), and ferritin (361.6±113.4 vs. 271.1±103.7, p<0.001) exhibited significant increases, while PaO₂ did not demonstrate a significant difference (p=0.858).

Correlational analysis

A correlation analysis was performed to assess the relationship between various clinical and biochemical parameters and troponin levels (Table 3). Although statistically significant, the correlations were weak to moderate with values (r=0.14-0.45), indicating modest associations.

**Table 3:** Correlational clinical and biochemical parameters analysis with troponin I levels. SOFA: Sequential Organ Failure Assessment; ICU: intensive care unit; HB: hemoglobin; WBC: white blood cells; RDW: red cell distribution width; BUN: blood urea nitrogen; ALT: alanine transaminase; AST: aspartate transaminase; CRP: C-reactive protein; SBP: systolic blood pressure; PT: prothrombin time

Variables	Correlation coefficient (r)	p-Value
CRP	0.149	0.036
ALT	0.204	0.004
AST	0.221	0.002
Lactate	0.246	<0.001
SOFA score	0.144	0.042
SBP	0.228	0.001
BUN	0.320	<0.001
D-dimer	0.163	0.021
Ferritin	0.181	0.010
PT	0.193	0.006
ICU stay	0.167	0.018
Hemoglobin	-0.252	<0.001
RDW	0.223	0.002
Neutrophils	0.452	<0.001

**Figure 5:**
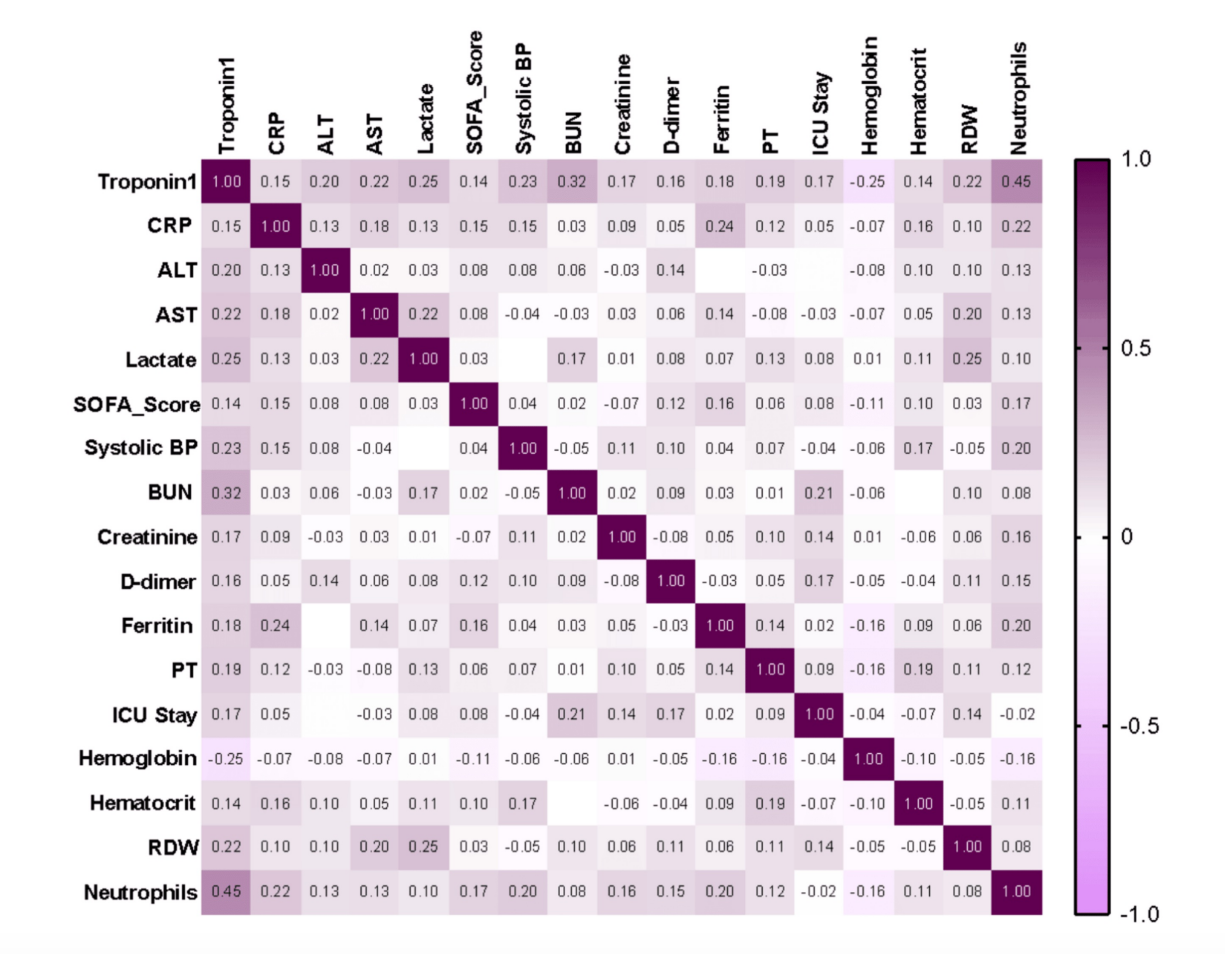
Correlational matrix of troponin I level with blood and biochemical parameters. SOFA: Sequential Organ Failure Assessment; BP: blood pressure; ICU: intensive care unit; HB: hemoglobin; WBC: white blood cells; RDW: red cell distribution width; BUN: blood urea nitrogen; ALT: alanine transaminase; AST: aspartate transaminase; CRP: C-reactive protein; PT: prothrombin time

Correlational analysis revealed a moderate association between troponin I levels and various markers indicative of systemic dysfunction in sepsis. Interestingly, troponin showed a positive correlation with neutrophil percentage, blood urea nitrogen (BUN), C-reactive protein (CRP), lactate, and RDW, indicating associations with immune activation, renal stress, inflammation, and hematologic dysregulation. Notable yet modest correlations were identified with liver enzymes (ALT, AST), coagulation markers (D-dimer, PT), and the duration of ICU stay. Inverse correlations were observed between hemoglobin levels and oxygen saturation. The results indicate that troponin I serves a complex role, reflecting not only cardiac injury but also the broader systemic disease burden associated with sepsis. The correlation matrix is shown in Figure 5.

Multivariate regression analysis

Through multivariate logistic regression, independent predictors of 28-day mortality in patients with sepsis were identified (Table 4). The model incorporated variables that are clinically significant or those that exhibited p<0.1 in univariate analyses.

**Table 4:** Multivariate logistic regression identifying independent predictors of 28-day mortality in patients with sepsis. SOFA: Sequential Organ Failure Assessment; BP: blood pressure; BMI: body mass index; ICU: intensive care unit; WBC: white blood cells; RBC: red blood cells; RDW: red cell distribution width; BUN: blood urea nitrogen; ALT: alanine transaminase; AST: aspartate transaminase; CRP: C-reactive protein; PaO_2_: partial pressure of oxygen in arterial blood; LDH: lactate dehydrogenase; APTT: activated partial thromboplastin time; PT: prothrombin time; CI (L): confidence interval lower; CI (U): confidence interval upper; OR: odds ratio

Variables	Adjusted OR	95% CI (L)	95% CI (U)	p-Value
Age	1.03	0.99	1.08	0.093
BMI	0.90	0.81	0.98	0.041
CRP	1.04	1.01	1.06	0.001
ALT	1.05	1.02	1.08	0.004
AST	0.97	0.93	1.01	0.108
PaO_2_	1.00	0.97	1.02	0.755
Lactate	1.23	0.73	2.08	0.432
SOFA score	0.98	0.92	1.05	0.597
Systolic BP	1.00	0.97	1.02	0.935
Heart rate	1.00	0.98	1.01	0.608
Respiratory rate	1.01	0.92	1.10	0.840
Temperature	0.96	0.64	1.43	0.838
Oxygen saturation	0.82	0.68	0.97	0.048
WBC	1.07	0.89	1.28	0.468
Procalcitonin	0.90	0.32	2.55	0.839
BUN	0.96	0.87	1.07	0.476
Creatinine	6.09	1.20	30.94	0.029
D-dimer	0.31	0.10	1.03	0.056
Ferritin	1.01	1.00	1.01	0.002
APTT	1.10	1.01	1.21	0.037
PT	0.89	0.72	1.10	0.275
Fluid resuscitation	1.03	0.59	1.79	0.924
ICU stay	0.98	0.92	1.04	0.460
Hospital stay	1.02	0.99	1.05	0.118
Hemoglobin	0.70	0.57	0.86	0.001
Hematocrit	0.97	0.88	1.06	0.475
Platelet	1.00	0.99	1.01	0.496
RBC	1.90	0.75	4.81	0.178
RDW	1.28	1.00	1.64	0.047
Neutrophils	1.05	1.00	1.10	0.040
Lymphocytes	0.88	0.80	0.97	0.010

Multivariate logistic regression revealed multiple independent predictors of mortality. Renal dysfunction, indicated by elevated creatinine levels; anemia, characterized by low hemoglobin; and systemic inflammation, as evidenced by elevated CRP and ALT, were significantly correlated with an increased risk of mortality. Coagulation markers, including elevated APTT and ferritin, along with neutrophilia, low lymphocyte count, low BMI, increased RDW, and reduced oxygen saturation, contributed independently. The findings underscore the complex factors contributing to sepsis-related mortality and support the prognostic value of troponin I alongside indicators of organ dysfunction, inflammation, and hematologic abnormalities.

Survival analysis

**Figure 6:**
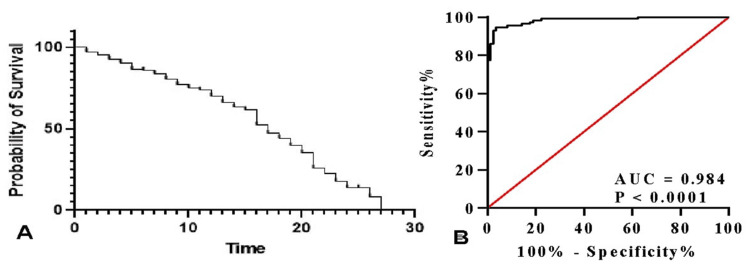
Kaplan-Meier survival analysis and ROC curve demonstrating the prognostic performance of troponin I for 28-day mortality. (A) Kaplan-Meier survival curve for 28-day mortality. (B) ROC curve to predict the prognostic efficiency of troponin. AUC: area under the curve; ROC: receiver operating characteristic

Kaplan-Meier analysis demonstrated a steady decline in survival probability over the 28-day follow-up among patients with elevated troponin levels (Figure 6A). ROC curve analysis confirmed excellent predictive performance of troponin I for mortality, with an area under the curve (AUC) of 0.986 (p<0.0001), underscoring its prognostic value in sepsis with organ dysfunction (Figure 6B).

## Discussion

This study reported that troponin can help predict sepsis outcomes, disease severity, inflammatory response, organ failure, and mortality. Elevated troponin levels were observed to be associated with poor outcomes as follows: more deaths occurred among troponin-positive patients, with longer ICU stays, and multiorgan dysfunction. These findings align with previous data and stress the forecasting importance of troponin in sepsis [11-13]. Although notable associations were identified, the observational nature of the study limits the ability to draw causal conclusions about the direct impact of troponin I on mortality related to sepsis. This study reported that patients who had troponin above the upper limit of normal had a 5.7-fold increased risk of death compared to patients with troponin in the normal range (RR=5.704, p<0.001). This is consistent with previous research that established an increase in troponin levels among septic patients as an independent factor in 28-day mortality in patients [12,14-17]. Lehner et al. found that troponin elevation represents not only myocardial injury but also systemic inflammation and microvascular dysfunction and serves as an independent determinant of sepsis mortality [16]. However, the study by Gajardo et al. found no significant association between troponin measurements and mortality among patients with sepsis [17].

This study also established a relationship between troponin concentration and the rise in SOFA scores. This research aligns with previous studies that found higher troponin levels in critically ill patients, which correlated with SOFA scores. This study underscores the additional prognostic value of cardiac biomarkers such as troponin I, contrasting with the Sepsis-3 criteria that focus on organ dysfunction through SOFA scores. Incorporating serial measurements or biomarkers like BNP and IL-6 could improve the accuracy of real-time sepsis risk stratification.

This study showed that troponin elevation had a significant correlation with essential biomarkers of inflammation, including CRP, ferritin, and D-dimer. Zheng et al. conducted a meta-analysis indicating that C-reactive protein (CRP) levels are elevated alongside troponin in septic patients. The cytokines associated with sepsis, particularly interleukin-6 (IL-6) and tumor necrosis factor-alpha (TNF-α), correlate with inflammation and myocardial dysfunction [12]. High CRP in conjunction with troponin may thus provide an early indication of adverse outcomes.

This study found a strong association between elevated troponin levels and increased D-dimer and ferritin levels, which are indicators of hypercoagulability and disseminated intravascular coagulation (DIC), respectively. These results highlight the relationship between inflammation, clotting abnormalities, and myocardial damage. Our results are consistent with previous studies reporting an association between elevated troponin in sepsis and various indices of coagulopathy and endothelial activation, including D-dimer and von Willebrand factor (VWF) [13,14]. This study’s raised D-dimer and prolonged PT/APTT indicate coagulopathy and disseminated intravascular coagulation (DIC), evident in sepsis with organ dysfunction. The relationship between elevated troponin levels and organ dysfunction in sepsis has been well-documented in numerous studies. However, some studies have reported findings that contradict this association. A study by Zheng et al. reported an association between elevated troponin levels and higher mortality rates in sepsis patients, yet failed to demonstrate a direct association between troponin values and markers of organ dysfunction, including BUN, creatinine, ALT, AST, or D-dimer. Elevated troponin is a mortality predictor but does not indicate the extent of septic organ dysfunction [12].

Lactate levels associated with anaerobic metabolism have been shown to be linked to myocardial hypoxia and sepsis severity, supporting the findings of the current study. The mean troponin level was significantly higher in patients requiring vasopressor support; these findings suggest that troponin may reflect subclinical myocardial dysfunction underlying septic shock [18]. Patients on vasopressors have higher troponin levels due to decreased cardiac output and perfusion [8].

Limitations of the study

This study has a few limitations. First, the sample size of the study was small. Sepsis has variable clinical manifestations across geographic, economic, and institutional contexts; thus, multicenter studies are needed to confirm these findings in diverse settings. Second, while multivariate regression was conducted, the observational design does not eliminate the possibility of residual confounding from unmeasured variables, including pre-existing cardiac dysfunction, varying treatment protocols, or time-to-intervention. Third, the study did not include serial troponin measurements and echocardiographic data for all patients. This may have offered additional insight into the dynamic relationship between myocardial injury and patient outcomes. While the ROC analysis demonstrated strong predictive accuracy, the lack of external validation could overestimate troponin's prognostic capabilities. Case identification involved initial bedside screening with final Sepsis-3 organ dysfunction inclusion, which may introduce heterogeneity. Mortality was high, reflecting a high-acuity case-mix. Furthermore, the investigation failed to consider pre-existing subclinical cardiac conditions that could affect baseline troponin levels, and there is a notable absence of external validation of these results in independent cohorts at this time.

Significance of the study

This research highlights the clinical significance of troponin I as a prognostic biomarker in sepsis. Troponin I indicates myocardial injury, systemic inflammation, and multiorgan involvement-essential elements of the septic cascade. Identifying patients at elevated mortality risk early in their hospital course allows troponin I to aid in risk stratification, improve clinical decision-making, and optimize resource utilization in ICU environments. In resource-constrained settings where sophisticated diagnostic tools may be scarce, serum troponin testing offers a straightforward and effective means to improve outcomes in patients with sepsis.

Future directions

Future research should investigate the effectiveness of serial troponin I measurements and their incorporation with additional biomarkers for real-time sepsis risk assessment. Multicenter validation and mechanistic studies are essential to ascertain whether troponin elevation indicates reversible myocardial dysfunction or irreversible injury. Given its robust prognostic accuracy, troponin I could be integrated into early sepsis management protocols to improve outcomes and inform long-term cardiovascular follow-up.

## Conclusions

This study emphasizes cardiac troponin I as a dependable and versatile biomarker in sepsis with organ dysfunction, indicating not just myocardial injury but also systemic inflammation, immune dysregulation, and multi-organ dysfunction. In addition to its known link to mortality, increased troponin I levels were associated with abnormalities in hepatic, renal, hematologic, and coagulation functions, highlighting its significance as a comprehensive marker of disease severity. Due to its accessibility and impressive prognostic accuracy, troponin I serves as a valuable addition to traditional scoring systems, such as SOFA, aiding in early risk stratification and informing critical care interventions. The results highlight troponin I as a significant yet often overlooked asset in the current approach to sepsis management.
